# Fluid mechanics of the left atrial ligation chick embryonic model of hypoplastic left heart syndrome

**DOI:** 10.1007/s10237-021-01447-3

**Published:** 2021-03-28

**Authors:** Sheldon Ho, Wei Xuan Chan, Choon Hwai Yap

**Affiliations:** 1 Department of Biomedical Engineering, National University of Singapore, Singapore, Singapore; 2 Department of Bioengineering, Imperial College London, London, UK

**Keywords:** Embryonic heart biomechanics, Hypoplastic left heart syndrome, Fluid mechanics, Chick embryonic left atrial ligation

## Abstract

Left atrial ligation (LAL) of the chick embryonic heart at HH21 is a model of the hypoplastic left heart syndrome (HLHS) disease, demonstrating morphological and hemodynamic features similar to human HLHS cases. Since it relies on mechanical intervention without genetic or pharmacological manipulations, it is a good model for understanding the biomechanics origins of such HLHS malformations. To date, however, the fluid mechanical environment of this model is poorly understood. In the current study, we performed 4D ultrasound imaging of LAL and normal chick embryonic hearts and 4D cardiac flow simulations to help shed light on the mechanical environment that may lead to the HLHS morphology. Results showed that the HH25 LAL atrial function was compromised, and velocities in the ventricle were reduced. The HH25 LAL ventricles developed a more triangular shape with a sharper apex, and in some cases, the atrioventricular junction shifted medially. These changes led to more sluggish flow near the ventricular free wall and apex, where more fluid particles moved in an oscillatory manner with the motion of the ventricular wall, while slowly being washed out, resulting in lower wall shear stresses and higher oscillatory indices. Consequent to these flow conditions, at HH28, even before septation is complete, the left ventricle was found to be hypoplastic while the right ventricle was found to be larger in compensation. Our results suggest that the low and oscillatory flow near the left side of the heart may play a role in causing the HLHS morphology in the LAL model.

**Supplementary Information:**

The online version contains supplementary material available at 10.1007/s10237-021-01447-3.

## Introduction

Hypoplastic left heart syndrome (HLHS), a type of severe CHD, represents 3% of CHD cases (Reller et al. 2008) but accounts for a disproportionate amount of neonatal deaths (Gilboa et al. 2010) (10.9%, highest mortality rate among all CHD) (Gilboa et al. 2010). HLHS is characterised by an underdevelopment of the left heart, accompanied by aortic valve stenosis or atresia, and hypoplastic ascending aorta and aortic arch (Franklin et al. 2017). Despite advances in medical diagnosis, little is known about the etiology of HLHS. HLHS is known to be heritable from parents with atrial-septal defect or ventricular-septal defect at 21 times the estimated population frequency (Nora et al. 1969) and is found to be multi-gene and genetically heterogeneous (Liu et al. 2017). However, known genetic causes are estimated to only account for 20% of CHD cases (Gelb and Chung 2014). It is thought that HLHS can be a result of abnormal hemodynamic loading because physical cardiac aberrations such as premature narrowing of the foramen ovale (Rychik et al. 1999), atretic aortic valve (Danford and Cronican 1992) or impaired ventricle function (Allan et al. 1989) are observed to precede the HLHS birth morphology.

In vivo studies of small animal models provided evidence that congenital malformations can be elicited by alteration of the mechanical flow environment (Tobita and Keller 2000; Kowalski et al. 2014; Tobita et al. 2002), and identified mechanobiological mechanism for such malformations (Duchemin et al. 2019; Groenendijk et al. 2005). It is thus important to understand the biomechanical environment of the embryonic heart, and understand what patterns of blood flow and forces may lead to the malformations.

Surgical left atrial ligation (LAL) of the chick embryonic heart is previously shown to be a good model for HLHS (Tobita et al. 2002). For this disease model, the ligation of left atrium (LA) at *embryonic day* (ED) 3.5, which corresponds to Hamburger-Hamilton stage (HH) 21, is found to reduce velocity and pressure of blood flow in the ventricle (Tobita et al. 2002). Eventually, aortic pressure is restored (Lucitti et al. 2005) while ventricular pressure remains depressed (Hu et al. 2009). At HH34, the left ventricle (LV) is found to be hypoplastic, while the right ventricle (RV) appears to have grown larger than normal in compensation (Sedmera et al. 1999), which are hallmarks of HLHS. At HH38, the LV is found to develop a subendocardial fibrous tissue with significant increase in collagen (Pesevski et al. 2018), suggesting the occurrence of fibroelastosis, similar to observations in human HLHS fetal hearts (McElhinney et al. 2010). Since the model is generated without pharmacological or genetic manipulation, it is a good model for demonstrating the biomechanics original of congenital cardiac malformations. Interestingly, performing right atrium (RA) ligation at HH34 in addition to LAL at HH21 is found to re-balance flow distributions to the two ventricles, enhance LV growth to rescue the HLHS morphology (deAlmeida et al. 2007), corroborating with the notion that abnormal flow is the cause of the malformations.

However, to date, the flow biomechanics of this disease model has not been well characterized. A previous study performed 2D microscopy flow visualization of flow streams within the LAL heart, and performed rigid wall flow simulations, and found that cardiac flow streams are altered, and that wall shear stresses (WSS) are reduced (Kowalski et al. 2014). However, flow in the embryonic heart is 4D in nature, and involves dynamic motions of various cardiac structures, and thus a more detailed investigation is necessary. For this reason, in this study, we performed careful and detailed 4D imaging and flow simulations of the normal and LAL chick embryonic heart to characterize organ dynamics and flow biomechanics. Emphasis was placed on the fluid dynamics at HH25 (ED4.5) as the causative factor, and on the anatomic differences at HH28 (ED5.5) to represent the resulting morphological growth responses.

## Materials and methods

### Animal model and imaging

All experimental work in this study was approved by the Institutional Animal Use and Care Committee of the National University of Singapore. Fertilized White Leghorn chicken eggs were sourced from a local farm and incubated blunt side up at 38 °C with approximately 60% humidity for 3.5 days. At ED3.5 (HH21), the eggs were placed under a stereoscope, a 1cm^2^ hole was made in the shell and the membranes removed to expose the embryo. The embryo was gently turned to the left-side up position, and a 10–0 polyamide suture was tied over the LA, effectively reducing the volume of the LA. Following that, the embryo was repositioned to the original right-side up position and the shell opening was sealed with Parafilm wrap.

Five normal and 4 LAL chick embryos at ED4.5 (HH25) and 4 normal and 4 LAL chick embryos at ED5.5 (HH28) underwent 4D imaging using high frequency ultrasound system (Vevo2100, Visual Sonics Inc., Canada) with the MS700 transducer (30–70 MHz). The scanning techniques adopted were according to prior studies conducted (Ho et al. 2017, 2019a; Tan et al. 2015). A sterilized polyurethane membrane was briefly placed over the exposed embryos to prevent direct contact of the embryo with the ultrasonic gel. The egg temperature was maintained with a custom heat pad with thermostat control. The embryo was mounted on a micro-translation stage and imaged at 50-µm intervals for 30–50 planes covering the entire heart, with each imaging plane producing a video of more than 20 cardiac cycles. The Doppler velocity of blood flow at the ventricle outlet was captured and smoothed to remove noise.

### Image processing

Using our previously published methods (Ho et al. 2017,2019a), raw ultrasound videos underwent temporal image correlation to matching phases over multiple cardiac cycles, to enable quadratic ensemble averaging of images from the multiple cardiac cycles to one cycle, which increased pixel intensities at blood spaces and reduced those at tissue spaces. Subsequently, spatial image correlation was performed to synchronize heartbeat timing neighbouring slices, such that images from different slices could be synchronized. 3D Segmentation of the cardiac blood spaces was performed at end-diastole using VMTK (www.vmtk.org), and then a validated cardiac motion estimation algorithm (Wiputra et al. 2020) was used to propagate the 3D reconstruction to all time points. Similar to previous work (Ho et al. 2019b), the threshold values for segmentation were iteratively determined so that volumetric calculations from motion tracking would show minimum retrograde flow across the atrioventricular junction, which was a feature demonstrated in the literature for both normal and LAL embryos (Tobita and Keller 2000), and which we could verify with Doppler measurements in some embryos.

### Area stretch ratio

To determine the contractility and cardiac wall deformational magnitudes, the area stretch ratio for the embryonic heart was calculated from the motion determined by the image tracking algorithm, using the same methods as in our previous publication (Ho et al. 2019b). This was calculated for all surface elements defining the embryonic heart reconstruction and was calculated from when each surface element was at its largest area, which was typically near to end-diastole, to when it was at its smallest area, which was typically near to end-systole. This measure was used instead of the actual myocardial stretch because the zero-stretch reference was not known. Complexity related to residual strains and stresses in the embryonic heart (Taber et al. 1993) created further difficulty in defining this reference state.

### Computational fluid dynamics simulations

Dynamic mesh flow simulations were performed using Fluent (ANSYS Workbench, Ansys Inc., PA, USA). Wall motions calculated from the motion tracking algorithm was input via user defined functions. The embryonic heart was meshed with a grid size of at least 400,000 cells, as determined via mesh convergence tests (Ho et al. 2019b). The simulation was conducted until cyclic convergence was achieved, such that the averaged WSS magnitude had less than 0.5% difference from the matching time point in the previous cardiac cycle.

The atrial inlet was prescribed with reference pressures from the literature (Keller et al. 1991). For LAL embryos, pressure magnitudes could be obtained from the literature (Tobita et al. 2002), but its temporal waveform could not. We thus used the waveform from normal chick embryos (Keller et al. 1991), adjusted to required magnitude. The ventricular outlet was extruded by about 2 diameters to remove boundary condition artefacts. Along the length of the extrusion, motion amplitudes were gradually decreased to zero via a sigmoid function. At the outlet end of the extrusion, uniform velocity boundary condition was imposed, and the velocity magnitudes was iteratively adjusted until velocities at the ventricular outlet boundary before the extrusion matched those from subject-specific Doppler measurements. In every iteration, flow simulation was conducted, and deviation of velocities at the ventricular outlet compared to Doppler measurements was calculated, as a ratio. This ratio was used to adjust the extrusion outlet boundary velocity magnitudes for the next iteration. Typically, 3–4 iterations were needed to reach a satisfactory match. This iterative adjustment was because the flow profile at the ventricular outlet was complex due to irregular cross-sectional shape and wall motion and flow dynamics, and could not be described by any idealized flow profile. Further, Doppler measurements could only provide the highest velocity within the flow profile, and not the profile itself.

From the results, WSS could be calculated as the viscosity multiplied by the velocity gradient in the surface normal direction, while oscillatory shear index (OSI) could be calculated as (He and Ku 1996):

1 $${\text{OSI}} = \frac{1}{2}\left( {1 - \frac{{\left| {\mathop \smallint \nolimits_{0}^{T} \vec{\tau } {\text{d}}t} \right|}}{{\mathop \smallint \nolimits_{0}^{T} \left| {\vec{\tau }} \right| {\text{d}}t}}} \right)$$

where $\tau$ was the WSS and *t* is time. Work done by the heart was calculated as.

2 $$W_{{{\text{ejection}}}} = \mathop \smallint \limits_{{{\text{systole}}}}^{ } \mathop \smallint \limits_{{{\text{surface}}}}^{ } P_{{{\text{wall}}}} *\left( {{\varvec{v}} \cdot \hat{\user2{n}}} \right) {\text{d}}A {\text{d}}t$$

where ${P}_{\mathrm{wall}}$ was the pressure at the endocardial wall, calculated from the literature absolute pressures and simulation intraventricular pressure gradients, ${\varvec{v}}$ was velocity, $\widehat{{\varvec{n}}}$ was the normal vector, and A was the surface area of cardiac structure,

### Statistics

Statistical testing was done by first confirming that the data distribution was normal via the Shapiro–Wilk test, before hypothesis testing via one-tailed t-test. One-tailed test is performed because we tested for differences with specific directionality.

## Results

### Effects of left atrium ligation on cardiac anatomy

**Fig. 1:**
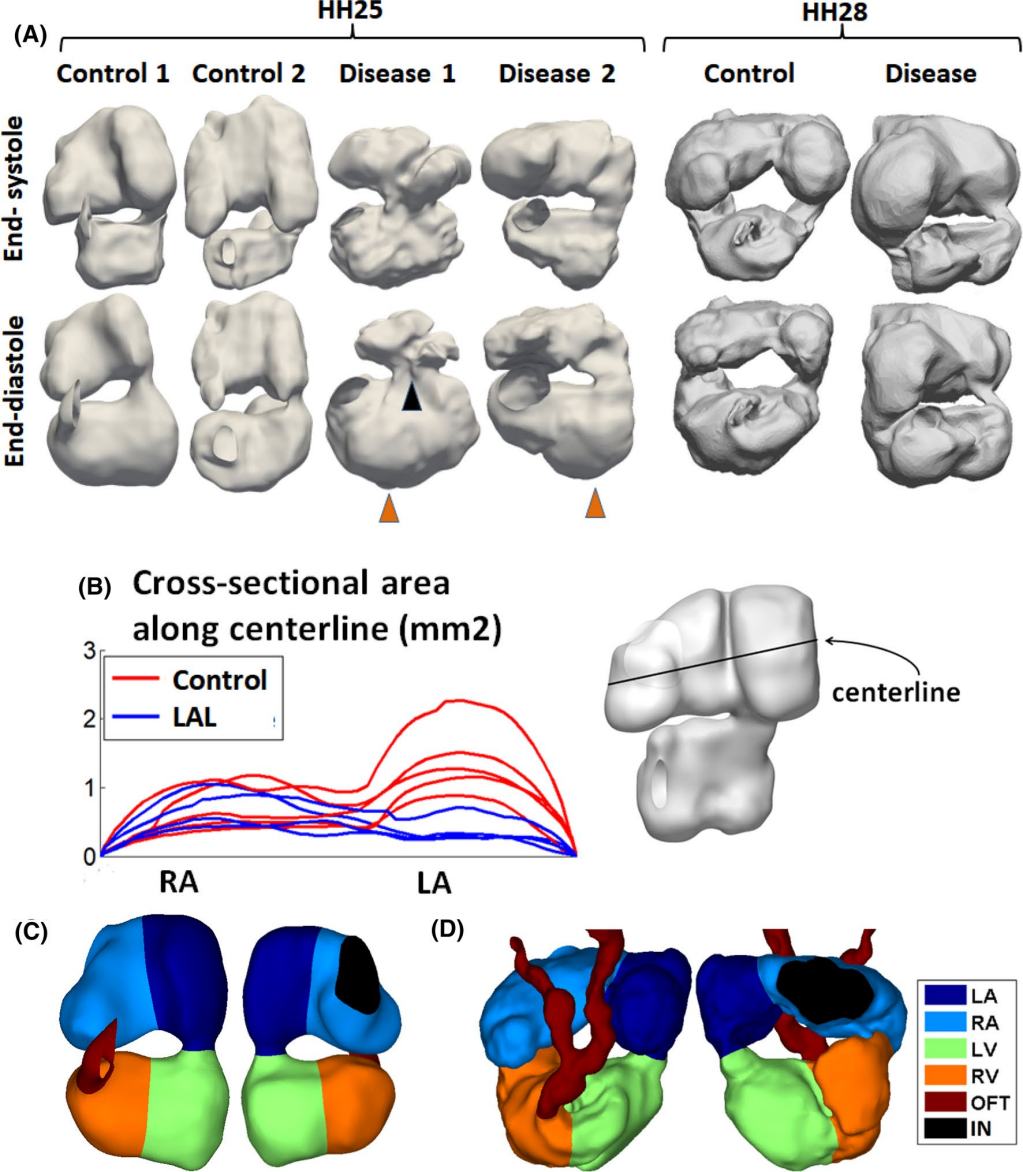
**a** Samples of reconstructions of the chick embryonic hearts blood space (other reconstructions are shown in supplementary Figs. 1 and 2). At HH25, there was only one atrio-ventricular junction, but by HH28, the atrio-ventricular junction divided into two. Left atrial ligation severely reduced left atrial volume, caused the ventricle to adopt a more triangular shape with a sharper apex (black arrow), and medially shifted the atrioventricular junction in some hearts (orange arrow, LAL1). **b** Cross-sectional area of the atrium along medial–lateral direction, during atrial end-diastole, demonstrating narrowing in the left atrium but not in the right atrium. **c**-**d** Delineation of the embryonic heart into the four cardiac structures for the **c** HH25 and **d** HH28 hearts. RA-right atrium, LA-left atrium, RV-right ventricle, LV-left ventricle, OFT-outflow tract, IN-sinus venosus inlet

**Fig. 2:**
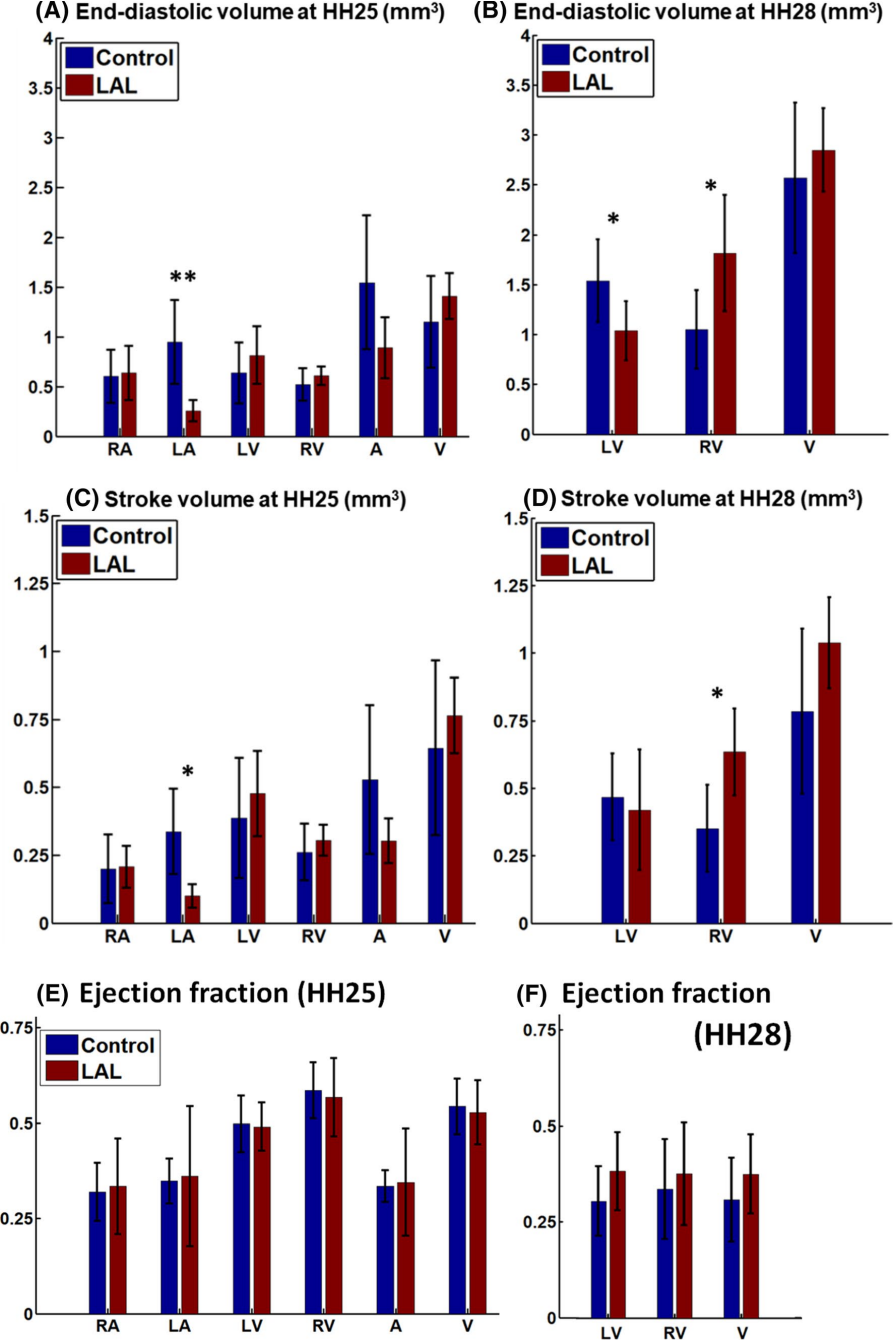
**a** Volumes, **b** stroke volumes, and **c** ejection fractions of the different cardiac structures at HH25 and HH28. RA-right atrium, LA-left atrium, RV-right ventricle, LV-left ventricle, A- the whole atrium (left and right combined), V- the whole ventricle (left and right combined). * denotes *p* < 0.05, ** denotes *p* < 0.01

3D reconstructions of the embryonic hearts are displayed in Fig. 1 and in supplementary text Figs. 1 and 2. LAL at HH21 affected both the size and the shape of the heart at HH25. Firstly, it significantly reduced the LA size (*p* < 0.001), as a direct consequence of the ligation, but did not alter the RA size (Fig. 1b). The narrowing of the LA was likely to increase resistance to flow moving from the RA through the LA into the ventricle. Secondly, HH25 LAL ventricles appeared more triangular compared to normal ventricles, having a sharper apex (Fig. 1a). Thirdly, LAL caused a medial shift in the atrioventricular junction in some cases (cases 1, 3 and 4), but not in the case 2, likely due to changes to the cardiac wall stresses imposed by the ligation.

The embryonic heart was divided into the four chambers, left and right atrium and ventricle, by manually tracing the developing septum. Since the septum protrudes into the luminal space, this would be equivalent to tracing along the narrowest site in the atria and in the ventricle. An example of the delineation for one HH25 and one HH28 hearts is shown in Fig. 1c and d, and in supplementary videos 1–2. This delineation was later used to quantify chamber volumes.

### Effects of left atrium ligation on cardiac function

The volumes and stroke volumes of the various cardiac structures are shown in Fig. 2. At HH25, LAL significantly reduced the ligated LA’s end-diastolic volume, by 73% as compared to controls (*p* < 0.05, Fig. 2a). However, volumes of other cardiac structures, including the RV, LV and RA, were not significantly different from the controls, although their mean volume values were generally higher. At HH28, however, the LAL LV end-diastolic volume became smaller than the controls end-diastolic volume while the LAL RV end-diastolic volume became larger than the controls (*p* < 0.05, Fig. 2a), suggesting that the LV was moving towards being hypoplastic, while the RV was growing larger to compensate for the smaller LV. While the average LV end-diastolic volume increased by 138% in the controls from HH25 to HH28, it had very little increase for LAL LV, increasing only 14% over the same period. While the average RV end-diastolic volume increased by 91% in the controls from HH25 to HH28, the increase almost doubled (increase of 181%) for LAL RV.

Atrium and ventricle stroke volumes were 0.528 ± 0.274 and 0.644 ± 0.321 mm^3^, respectively, for control cohort, and 0.303 ± 0.082 mm^3^ and 0.764 ± 0.138 mm^3^ for LAL cohort (Fig. 2b). LAL significantly reduced the atrial-ventricular stroke volume ratio from 0.87 ± 0.36 to 0.42 ± 0.16 (*P* < 0.05). Since the atrial stroke volume was smaller than the ventricular stroke volume, some blood flowed directly from sinus venosus into the ventricle without the aid of the atrial pumping. We previously reported this “through-flow” phenomenon for normal chick embryonic hearts (Ho et al. 2019b). In the current LAL cohort, this phenomenon was more significant, since the reduction in LA function with LAL extended the stroke volume differential between the atria and ventricle.

Ejection fraction quantifications are shown in Fig. 2e and f. No significant differences were observed for any cardiac structure for both HH25 and HH28.

### Effects of left atrium ligation on motion and contraction dynamics

**Fig. 3:**
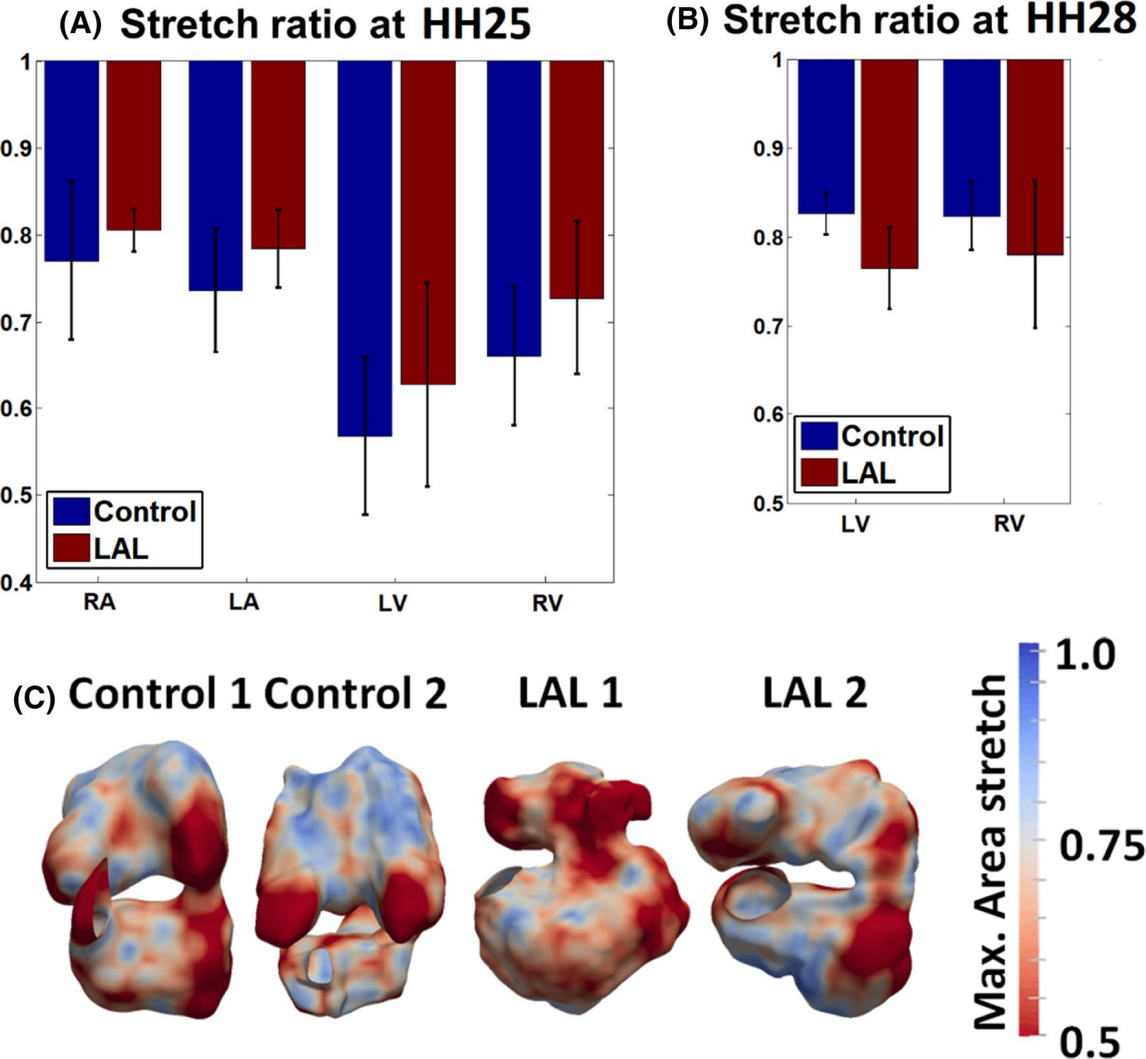
**a** Diastole-to-Systole wall area stretch ratios for **a** HH25 and **b** HH28 hearts. **c** Spatial pattern of maximum area stretch ratios

The area stretch ratio of the surface of our embryonic heart reconstruction from end-diastole to end-systole was used as a gauge of the contractile dynamics of the heart, and was plotted in the spatially averaged form in Fig. 3. Although the mean stretch magnitude was higher for the LA and RA at HH25 and for the LV and RV at HH25 with LAL, these differences were not significant.

### Effects of LAL on cardiac fluid dynamics

**Fig. 4:**
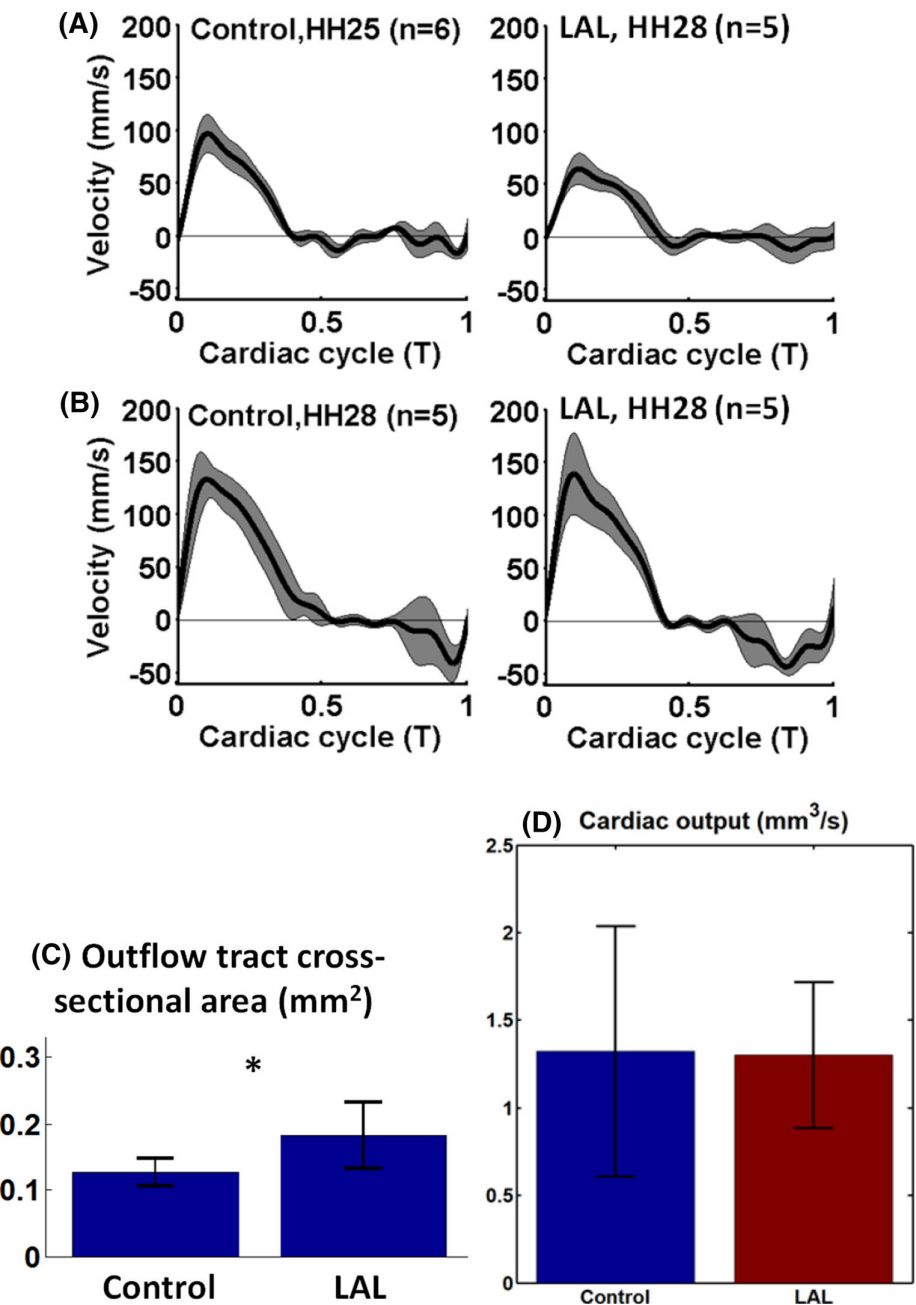
**a**, **b** Pulsewave Doppler measurement of velocity at the proximal outflow tract with standard deviation demarcated in grey, for **a** HH25, control (*n* = 6) and LAL hearts (*n* = 5) and **b** HH28, control (*n* = 5) and LAL hearts (*n* = 5). Peak velocity for HH25 was significantly different between control and LAL (*p* < 0.01) **c** outflow tract for control and LAL (*P* < 0.05). **d** Cardiac output of the HH25 embryonic hearts

Doppler velocity measurements revealed that at HH25, the LAL hearts had a similar ventricular outflow velocity waveform as control hearts, but there was a decrease in the maximum flow velocity (*p* < 0.01, Fig. 4). Interestingly, at HH28, LAL hearts recovered their outflow velocities to be similar to that of control hearts. These observations corroborated with previous literature (Lucitti et al. 2005). Further, HH28 LAL Doppler waveforms showed longer durations of retrograde velocities during ventricular end-diastole, but otherwise, the waveforms looked similar to those of normal hearts.

To test whether the reduced outflow velocity in HH25 LAL hearts were due to decreased volume flow rate or due to dilation in the outflow tract (OFT), we analysed the outflow tract lumen area, during ventricular systole. We found that the LAL OFT was found significantly wider than control OFT (*p* < 0.05, Fig. 4c). Given that the HH25 LAL ventricular had stroke volumes that was not reduced from controls (Fig. 2b), we concluded that flow rates were not reduced, and lower flow velocities were likely due to dilated OFT. Indeed, cardiac output calculations (Fig. 4d) showed that both LAL and control groups have the same cardiac output.

**Fig. 5:**
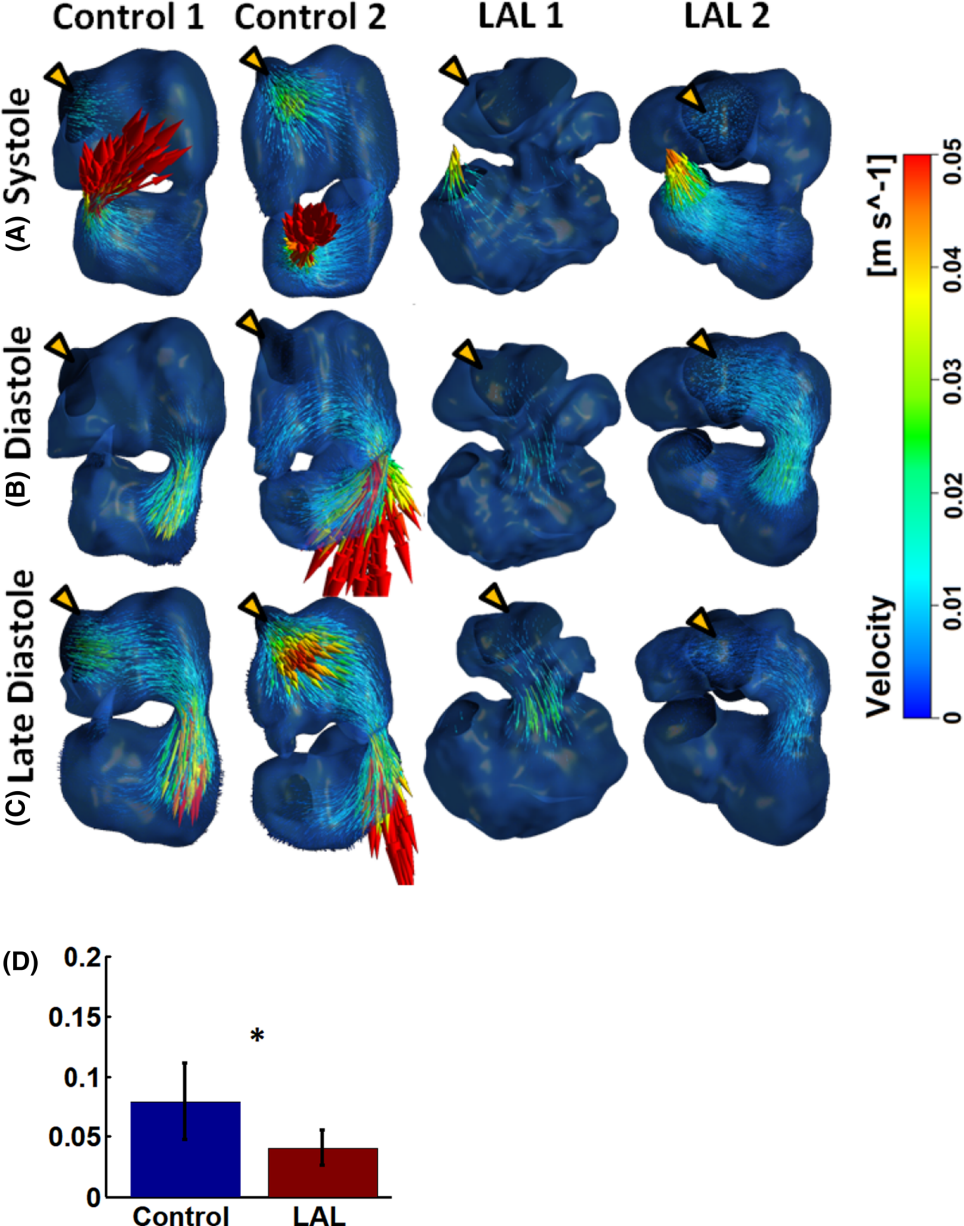
Velocity flow field of some embryonic hearts at HH25. Other samples are shown in supplementary Fig. 3. **a** Contraction of the ventricle during systole pumps blood out into the outflow tract. **b** Expansion of the ventricle draws blood from the atria through atrioventricular junction with aid of atria contraction. **c** Atrial contraction expired, leaving the ventricle to draw its inflow directly from the sinus venosus. “Through-flow” or flow moving from the veins towards the ventricle without atrial pumping aid occurs. Yellow arrow indicates atrial inlet. **d** Temporal peak of the volume-averaged velocity in the ventricle during diastole. * *p* < 0.05

HH25 flow field results from the flow simulations are shown in Fig. 5, Supplementary Fig. 3, Supplementary videos 3–4 for control hearts, and Supplementary videos 5–6 for LAL hearts. Flow results showed similarities and differences between normal and LAL hearts. In terms of similarities, firstly, both LAL and control heart had laminar flow due to the low Reynolds number, which was less than 10 in the atria and ventricle, and less than 25 at the atrioventricular junction and outflow tract. Consequently, little secondary flow features were observed. Further, in both types of hearts, velocities were the highest at the atrioventricular junction and the outflow tract outlet, since these were the narrowest points, and occurred in the form of flow jets.

In terms of differences, firstly, there was a weaker cooperation between the atria and ventricle during ventricular filling. In control hearts, ventricle diastolic expansion occurred mostly in conjunction with atrial contraction, but during late diastole, contraction of atrium ceased earlier than the expansion of the ventricle, resulting in a “through-flow” pattern where the ventricle drew blood directly from sinus venosus, without the aid of atria pumping (shown in Fig. 5c). In the LAL hearts, atrium stroke volume was reduced compared to controls, and the cessation of atrial contraction occurred earlier, resulting in prolonged periods of this “through-flow” pattern. Secondly, diastolic velocities in LAL ventricles tended to be lower, as can be demonstrated by the peak diastolic volume-averaged velocity in the ventricle in Fig. 5d. This was likely caused by weakened atrial function, and altered flow patterns due to geometric changes to the LAL ventricle, such as the weak and oscillatory flow pattern that will be discussed below.

**Fig. 6:**
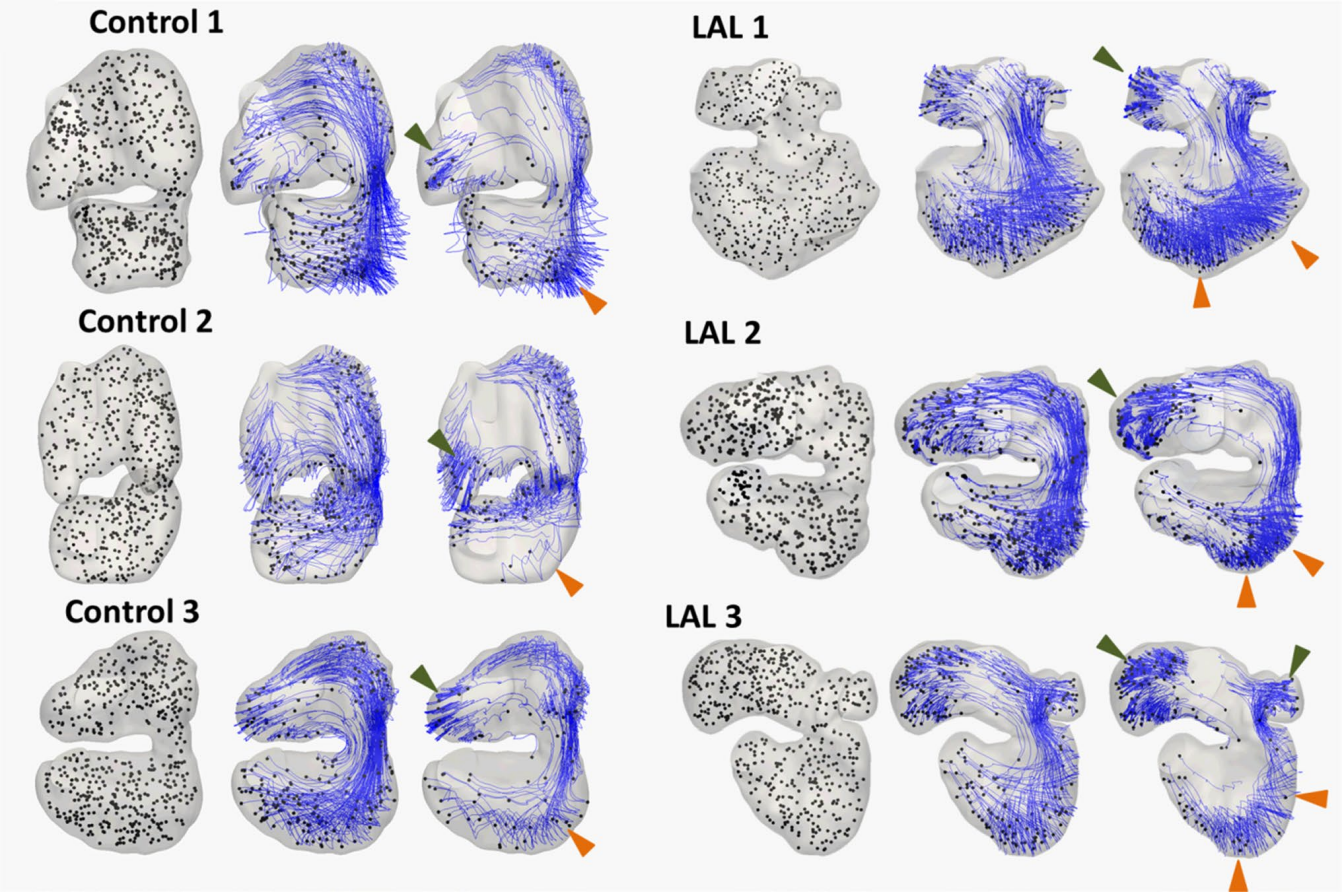
The pathlines of particles in the embryonic heart after 0, 2, and 5 cardiac cycles for HH25 three hearts. Green arrow demarcates region of high particle retention in the atrial appendage. Orange arrow demarcates region of high particle retention in ventricle apex and left ventricular free wall. Please refer to supplementary video 6–9 for advection of particles, as these videos can display particle motions much better

To further understand differences in the flow dynamics, 500 particles were seeded randomly throughout the embryonic hearts and motion tracked over 5 cardiac cycles, assuming that the particles adopted the same velocity as the fluid at its location, and that particles were negligibly small such that their presence would not change the flow field. The criterion for particle seeding was that they were not too close to the wall (> 1e-6 m), as otherwise, they would have too little motion for meaningful tracking. Tracking results are shown in Fig. 6a and in supplementary videos 7–10. In both control and disease model cases, some particles were found to have high retention in the left free wall and the apex of the ventricle. These fluid particles were sufficiently far away from the main flow streams, and had lower velocities, and bobbed around with the contractile motion of the heart in an oscillatory flow pattern, while being washed out more slowly. In LAL ventricles, however, the number of such particles was enhanced. Blood flow at the LV free wall was more sluggish, and less influenced by forward flow streams. The oscillatory nature of flow in the LAL LV was thus enhanced.

This difference was likely caused by a few factors. Firstly, the lower flow velocities in the HH25 LAL ventricle, resulting in reduced washout momentum for ventricular fluid. Secondly, in 3 LAL hearts, the atrioventricular junction had a medial shift, resulting in the strong atrioventricular flow becoming further away from the left ventricular free wall, again to reduce washout. Thirdly, since the LAL hearts had a sharper apex, fluid in the apex became more shielded from the fast moving forward flow and led to slower washout.

We previously reported that the atrial appendages experience high fluid particle retention compared to the rest of the heart, as the elongated atrial appendages act as shelters for blood in the atrial appendages and shielding them from the main flow stream (Ho et al. 2019b). Here, we observed that the same still applied to the LAL right atrial appendage. The left atrial appendage could not be clearly delineated due to the ligation.

### Effects of the LAL on flow biomechanical force environment

**Fig. 7:**
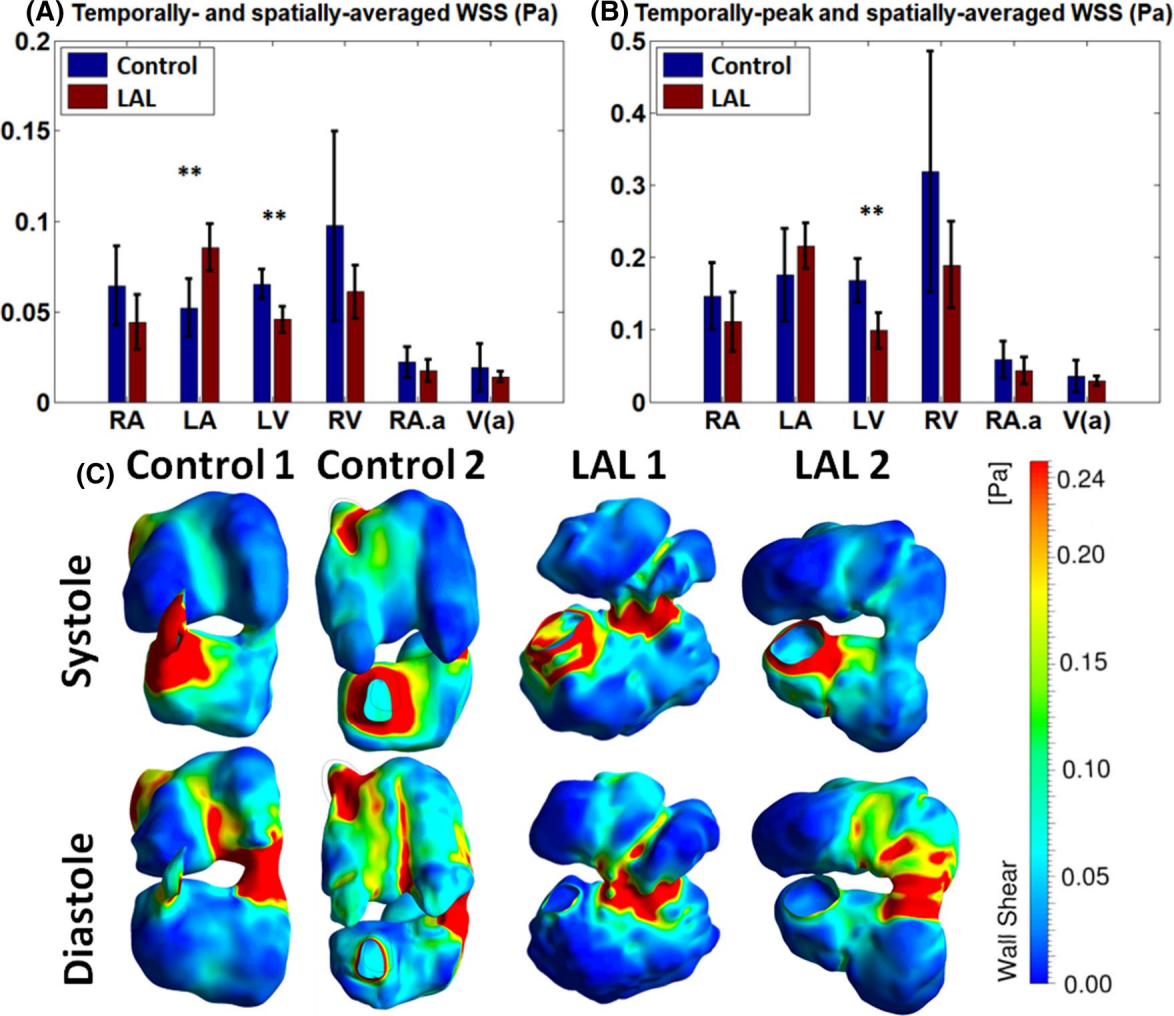
**a** Temporally and spatially averaged wall shear stress (WSS), and **b** temporally peak and spatially averaged WSS in the different cardiac structures at HH25. **c** Spatial pattern of WSS in some heart samples. Other samples are shown in Supplementary Fig. 5. RA—right atrium, LA—left atrium, RV—right ventricle, LV—left ventricle, RA.a—right atrial appendage V(a)—ventricular apex. ** *p*-value < 0.01

Flow simulation results showed that at HH25, both LAL and control hearts experienced high spatial and temporal variation of flow WSS due to the complex anatomy of the heart and its dynamic motions, as shown in Fig. 7c, supplementary Fig. 4, and videos 11–14. Generally, WSS were similarly elevated at region with narrower lumen such as the atrioventricular junction and outflow tract, and at the mid-line of atria and mid-line of the ventricle. At these midline regions, septation growth protruded into the blood fluid space to create narrower channels and higher velocity, and together with the high surface curvatures on the septum, this caused higher WSS at these regions.

**Fig. 8:**
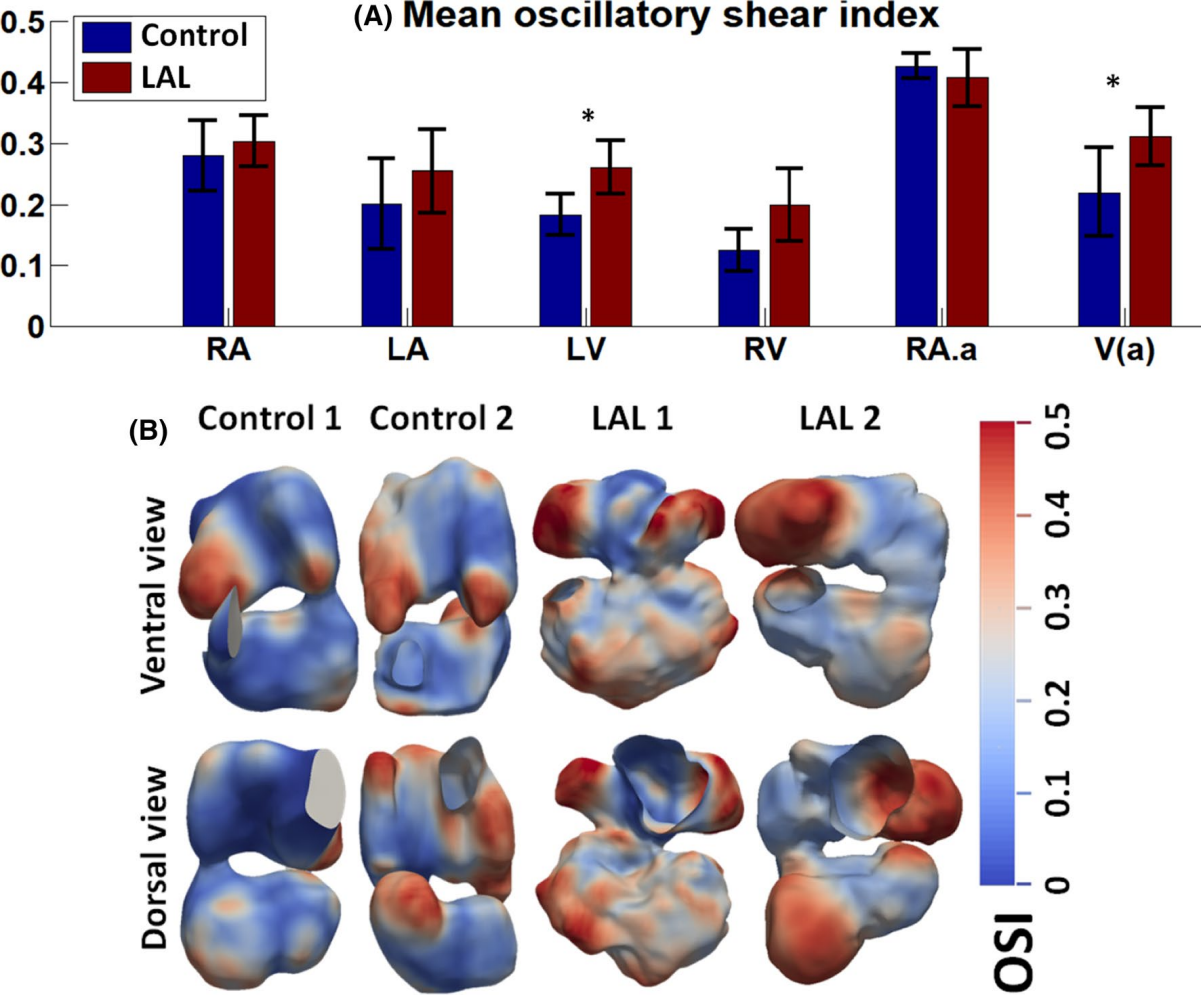
**a** Spatially averaged oscillatory shear index (OSI) in the different cardiac structures for HH25 hearts. **b** Spatial pattern of some embryonic hearts. RA-right atrium, LA-left atrium, LV-left ventricle, RV-right ventricle, AV-j – atrioventricular junction, RA.a-Right atrial appendage, V(a)-Ventricle apex. * *p* < 0.05, ** *p*-value < 0.01

Mechanical forces exerted on the HH25 LAL heart shared similar patterns with control hearts but were different in WSS magnitudes and oscillatory shear amplitudes in some cardiac structures (Figs. 7 and 8). Figure 7 shows that in the LAL LV, WSS (both temporal-average and temporal peak WSS) was significantly decreased from normal hearts (*p* < 0.01). This was likely due to the reduced velocities in the LAL ventricle, and the sluggish flow near the LV free wall and apex, as discussed above. Figure 7 further shows that in other regions of the heart except for the LA, WSS also generally decreased in LAL hearts, but differences from normal hearts were not statistically significant. In the LA, because LAL caused a narrowing of the LA, WSS was increased, and the temporal- and spatial-averaged WSS showed statistical significance (Fig. 7, *p* < 0.01).

Figure 8a shows the spatial-averaged OSI for various cardiac chambers at HH25, while Fig. 8b shows the spatial distribution of OSI. Generally, spatial patterns were similar between LAL and control hearts, where OSI was higher in the RA appendage, and the LV free wall. However, OSI magnitude was significantly higher in both the LV and RV and the apical region in the LAL heart compared to control hearts (*p* < 0.05, Fig. 8a). These results were most likely a consequent of the higher particle retention time in these areas, and the sluggish and oscillatory nature of the flow there, as described above.

### Effects of LAL on ejection work done

**Fig. 9:**
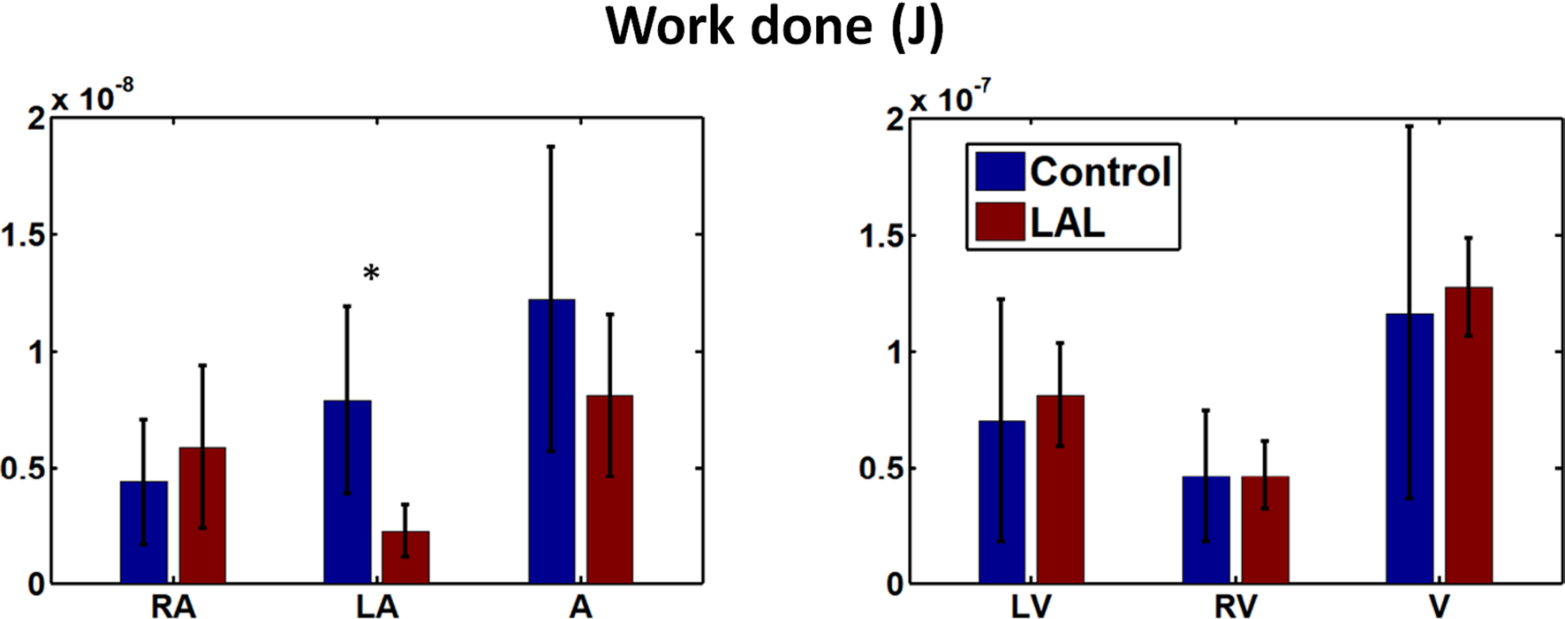
Work done by the embryonic heart in different regions of embryonic heart during contraction for the HH25 heart. * *p* < 0.05

In terms of work done by the HH25 heart, LAL resulted in changes in the atrium, but not in the ventricle (Fig. 9). In LAL hearts, LA work done was significantly decreased, while that for the RA was significantly increased. The reduced work done by the LAL LA was likely due to the smaller size (Fig. 2a) and thus smaller motion. For the LAL RA, on the other hand, size was not reduced (Fig. 2a), however, the narrowed LA (Fig. 1b) posed greater resistance to flow, thus requiring the RA to increase work done to push blood through to the ventricle. However, despite this compensation by RA, work done by the whole LAL atrium was still reduced as compared to control. As for the ventricle, despite changes to the WSS and OSI after LAL, RV and LV work done did not change.

## Discussion

In the current study, we conducted an investigation of the fluid mechanics in the chick embryonic heart model of HLHS, achieved via LAL, as a means of discovering flow biomechanics which could be contributing to this disease. We tapped into robust, previously established techniques so as to give high level of details. The use of high-frequency ultrasound for in vivo 4D scans allowed us to capture 3D anatomy and motion dynamics to aid the assessment of chamber volumes and cardiac function. This improved on previous work performing 2D scans of the LAL hearts (Tobita et al. 2002), and due to the large and complex changes to the cardiac geometry after LAL, such 2D scans might be insufficiently accurate. Our approach has also enabled full 4D flow simulations for the LAL chick embryonic hearts with full consideration of the cardiac motion dynamics, which improved on previous work that did not consider the motion (Kowalski et al. 2014).

Our investigations showed that the LAL hearts were not significantly remodelled at HH25, but had begun remodelling towards a hypoplastic LV and an enlarged compensated RV at HH28, when septation was still quite far from completion. Our observations could be corroborated by previous studies, which reported that at HH27, LAL LV was not detectably smaller (Tobita and Keller 2000), but by HH29, RV dilation and reduced LV length could be observed (Sedmera et al. 1999). A prevailing hypothesis for pathogenesis of the HLHS morphology was that the ligated LA led to reduced LV volume load after septation to cause the morphology (Sedmera et al. 1999). Our observation of remodelling towards HLHS morphology at an earlier time point, even before septation was complete, however, challenged the idea that reduced volume load was the sole contributor to the disease morphology, and suggested that altered flow conditions before septation had already begun stimulating the mal-development. We had thus gone into detailed investigation of the fluid mechanics of the HH25 LAL heart, at the embryonic stage slightly before our morphology observation above. Our basis was that morphological changes would not occur immediately, but would take some time after the alteration of flow stimuli to occur.

From our investigation of HH25 embryonic fluid mechanics, we found that there were reduced velocities within the ventricle and at the outflow tract, reduced WSS and elevated oscillatory flow patterns at the LV free wall and ventricular apex, and higher fluid particle retention in the LV. Since WSS (Groenendijk et al. 2005; Sidhwani and Yelon 2019) and the oscillatory nature of WSS (Sidhwani and Yelon 2019; Boselli et al. 2017) are known to influence the embryonic heart development, these observed differences could explain the altered morphological remodelling in LAL hearts by HH28. In our study, due to an absence of genetic and pharmacological modulation, it was likely that flow biomechanics were the cause of the mal-development. Since embryonic oxygenation is mainly via diffusion at these stages, we propose that it was unlikely that mal-development is due to changes in oxygenation (Burggren 2004).

Our results further suggested that morphological and organ dynamics changes were the cause of these altered flow patterns and forces in the HH25 ventricle. The disrupted LA function reduced atrial stroke volume (Fig. 2c), making it less able to aid ventricular filling, and the narrowed LA (Fig. 1b) posed additional flow resistance. These factors were likely to contribute to the observed reduced diastolic ventricular velocities (Fig. 5d), which is corroborated by literature (Tobita and Keller 2000).

Further, the alteration in ventricular shape was such that it adopted a more triangular shape with a sharper apex (Fig. 1a), and this resulted in fluid near to the apex region being further away from faster moving flow streams. In some HH25 LAL hearts, the atrioventricular junction was found to have shifted medially (Fig. 1a), likely due to the mechanical forces imposed by the atrial ligation. This had resulted in fluid in the LV free wall to be further away from fast moving flow streams, again resulting in lower velocities (Fig. 6).

The reduction in flow velocities and the situation where larger regions of fluid at the LV endocardial surface were further away from high velocity regions had consequently resulted in the wall shear stresses in the LV to be low and oscillatory (Figs. 7 and 8). We propose that these altered flow stresses would then stimulate altered mechanobiology in the LV to contribute to the HLHS morphology. However, we further propose the interesting possibility that the altered flow patterns and forces could have led to an alteration in the septation location, to result in a smaller LV and larger RV, given that cardiac cushions are mechanosensitive and rely on mechanical cues for their formation (Bartman et al. 2004; Hogers et al. 1997).

We noted, however, that although flow velocities were observed to be reduced in LAL hearts, our volume measurements did not show a decrease in the stroke volume of the HH25 LAL ventricle, suggesting that there was no decrease in volume flow rate. Calculations of cardiac output from our simulations further showed that they were similar between LAL and control groups. Reduced flow rate was thus not a contributing factor to observed changes in flow dynamics above. Even though our Doppler measurements at the OFT showed decreased velocities in the HH25 LAL heart, we found that the OFT had dilated, and this compensatory effect had prevented volume flow rates and stroke volume from being lower than control hearts. Our findings with Doppler measurements of decreased velocities at HH25 after LAL, but restoration of velocities to similar to control hearts at HH28, agreed with previous studies (Tobita and Keller 2000; Lucitti et al. 2005). However, we caution that using velocity measurements alone, or in concert with 2D scans, might have limitations in accurately representing volume flow rates or cardiac stroke volumes.

## Limitations

First, data from the disease models were found to have higher variability, which could be due to variability in the LAL procedure, given that this was a delicate procedure that was difficult to perform in a highly precise manner. Second, the amount of time required for image processing, motion modelling and simulation of flow dynamics were substantial for each sample, and limited our experimental sample sizes. Finally, our high-frequency ultrasound did not have sufficient resolution to properly visualize trabeculation. Thus, the ventricle trabeculation was removed during the segmentation process. Our flow modelling thus represented the macroscopic flow features, and future work should investigate how these macroscopic features can translate to localized WSS and pressures on endocardial cells.

## Conclusions

Left atrial ligation was found to be capable of altering the mechanical flow environment in the embryonic heart at HH25, resulting in lower flow velocity and WSS while elevating OSI at ventricle apex and left free wall of the ventricle. The changes in biomechanical stimuli could have led to the hyperplastic right ventricle and hypoplastic left ventricle that were observed in LAL embryonic heart at HH28, even before septation is completed.

## Supplementary Information

Below is the link to the electronic supplementary material.

Supplementary file1 (DOCX 4097 KB)

Supplementary file2 (MP4 153 KB)

Supplementary file3 (MP4 209 KB)

Supplementary file4 (MP4 251 KB)

Supplementary file5 (MP4 512 KB)

Supplementary file6 (MP4 388 KB)

Supplementary file7 (MP4 1228 KB)

Supplementary file8 (MP4 2213 KB)

Supplementary file9 (MP4 1594 KB)

Supplementary file10 (MP4 2222 KB)

Supplementary file11 (MP4 1563 KB)

Supplementary file12 (MP4 487 KB)

Supplementary file13 (MP4 363 KB)

Supplementary file14 (MP4 618 KB)

Supplementary file15 (MP4 616 KB)
