# Supplementary material for: Fluid mechanics of the left atrial ligation chick embryonic model of hypoplastic left heart syndrome
Source: Biomech Model Mechanobiol. 2021 Mar 28;20(4):1337–51. doi: 10.1007/s10237-021-01447-3 (PMC8298253; doi:10.1007/s10237-021-01447-3)
Supplement: Supplementary file 1 — Supplementary file1 (DOCX 4097 KB) [file 10237_2021_1447_MOESM1_ESM.docx]

# Supplementary Figures


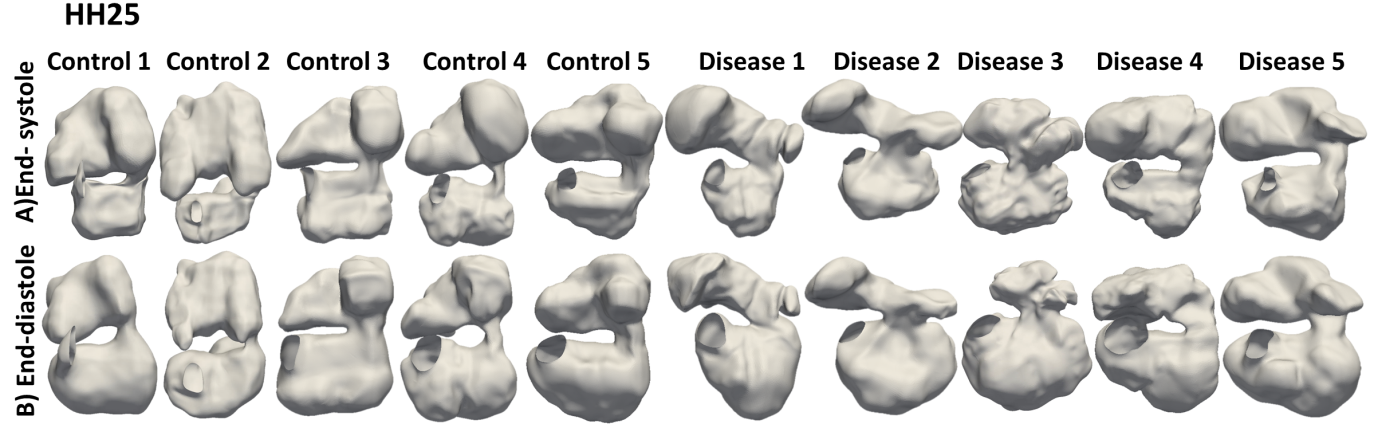


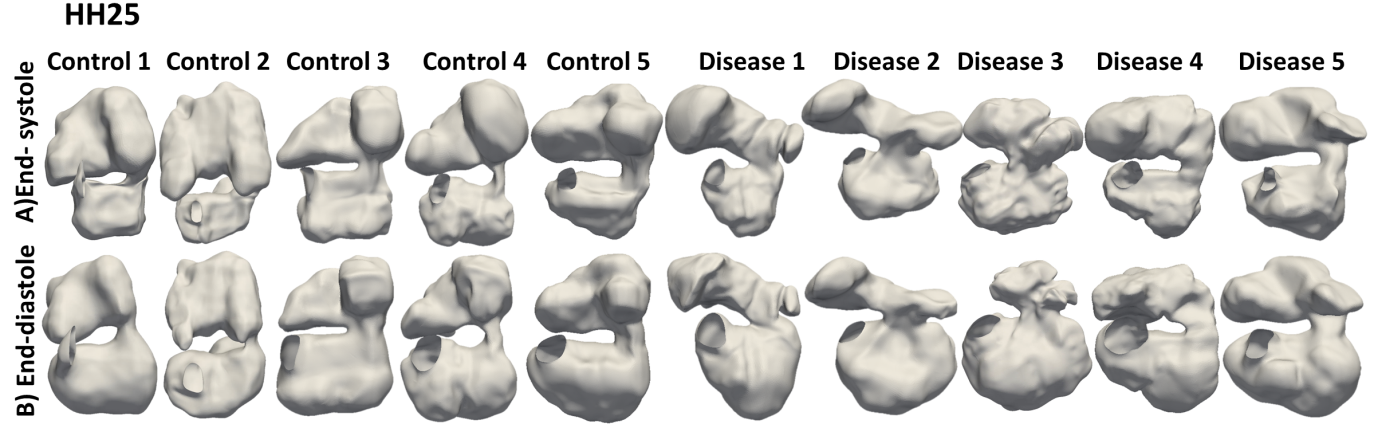

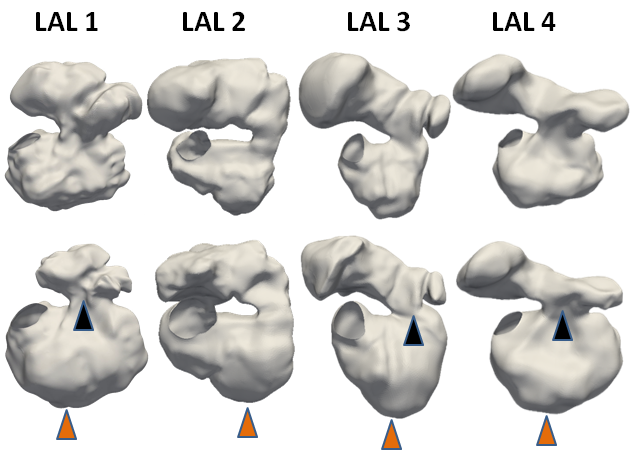


**Supplementary Figure 1: reconstructions of the blood volumes of the chick embryonic hearts at HH25. (A) Normal hearts. (B) Left atrial ligated hearts. Left atrial ligation severely reduced left atrial volume, caused the ventricle to adopt a more triangular shape with a sharper apex (orange arrow), and medially shifted the atrioventricular junction in some hearts (black arrow).**


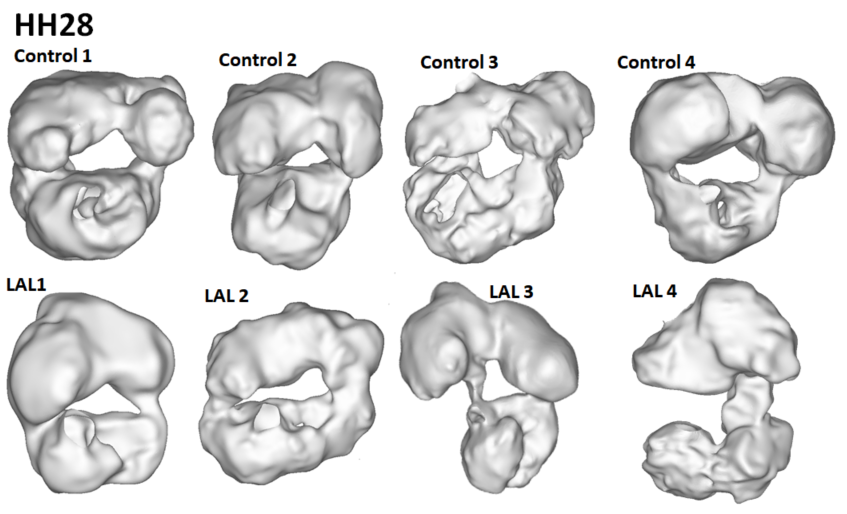


**Supplementary Figure 2: reconstructions of the blood volumes of the chick embryonic hearts at HH28.**


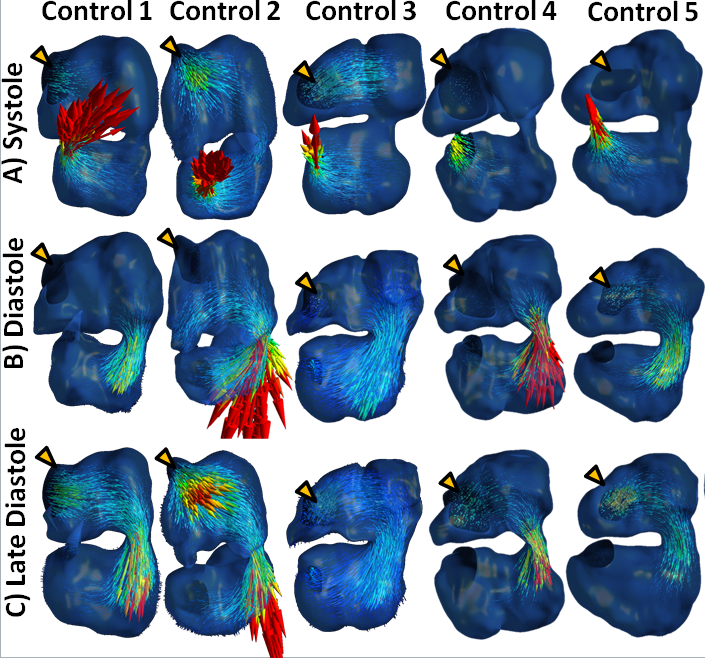


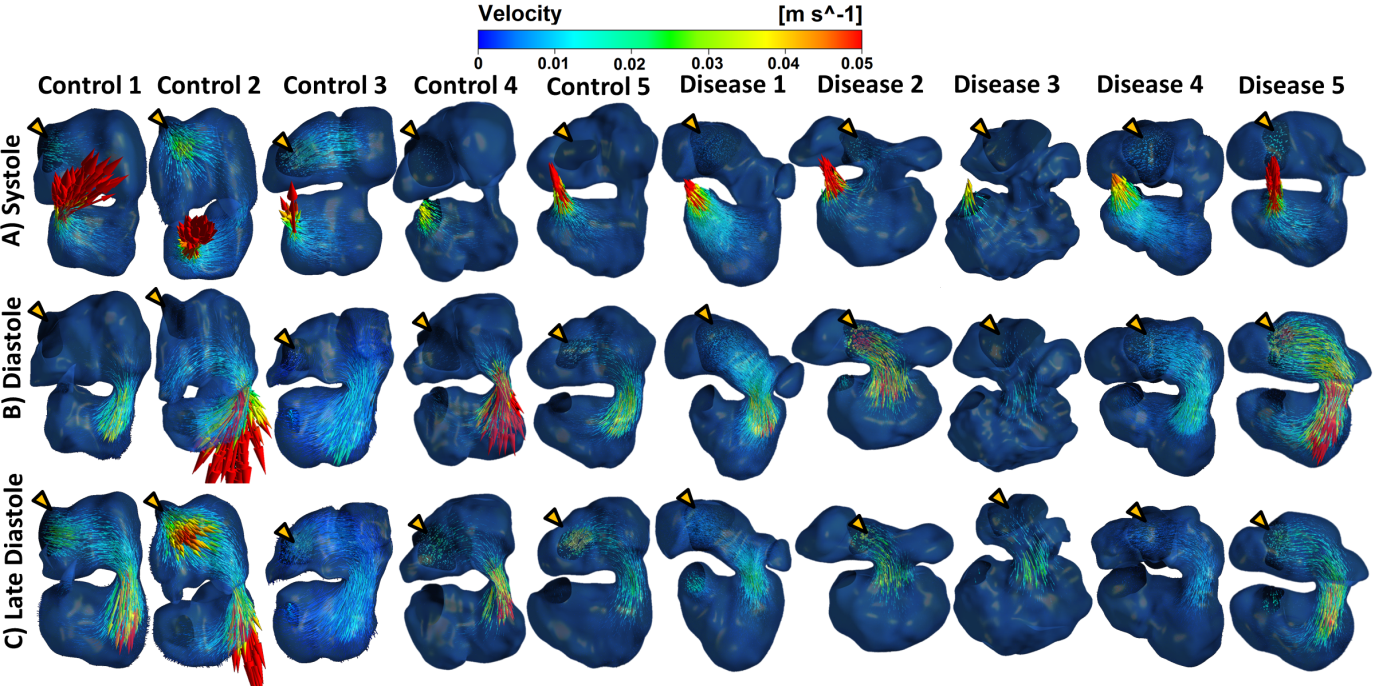

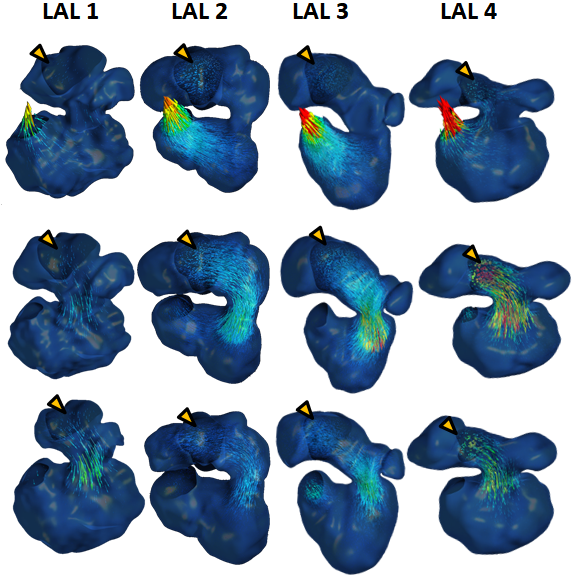

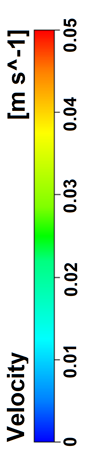


**Supplementary Figure 3: Velocity flow fields of all embryonic heart samples at HH25. (A) Contraction of the ventricle during systole pumps blood out into the outflow tract. (B) Expansion of the ventricle draws blood from the atria through atrioventricular junction with aid of atria contraction. (C) Atrial contraction expired, leaving the ventricle to draw its inflow directly from the venous network. “Through-flow” or flow moving from the veins towards the ventricle without atrial pumping aid occurs.**


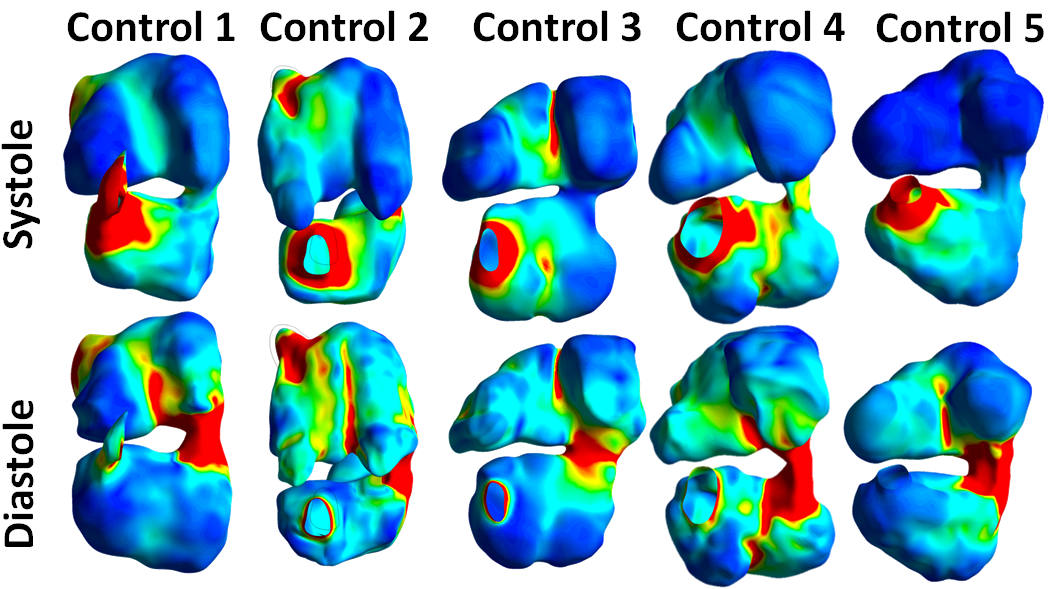


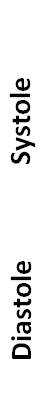

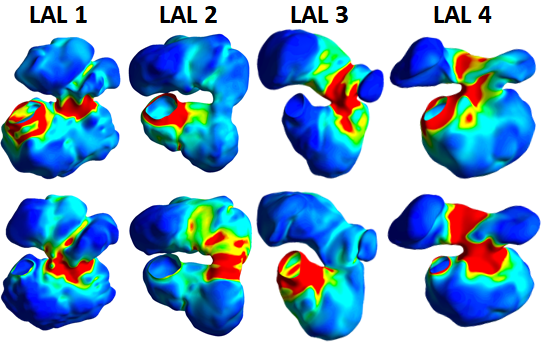
**
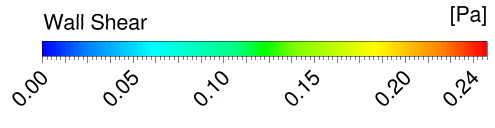
**

**Supplementary Figure 4: Spatial pattern of WSS of chick embryonic hearts at HH25, at systole and diastole.**
